# The case report of MOG and NMDAR IgG double positive encephalitis treated with subcutaneous ofatumumab

**DOI:** 10.3389/fimmu.2023.1183488

**Published:** 2023-08-15

**Authors:** Rui Zhang, Li Wang, Yongli Tao, Xiaofeng Zhang, Kai Liu, Bo Song, Yuming Xu

**Affiliations:** ^1^ Department of Neurology, The First Affiliated Hospital of Zhengzhou University, Zhengzhou, Henan, China; ^2^ Henan Key Laboratory of Cerebrovascular Diseases, The First Affiliated Hospital of Zhengzhou University, Zhengzhou University, Zhengzhou, Henan, China

**Keywords:** isolated seizures, N-methyl-D-aspartate receptor, myelin oligodendrocyte glycoprotein, autoimmune encephalitis, overlap syndrome, ofatumumab

## Abstract

The phenotypic spectrum of myelin oligodendrocyte glycoprotein (MOG)- IgG–associated disorders (MOGAD) has broadened in the past few years, and atypical phenotypes are increasingly recognized. Isolated seizures and MRI-negative brainstem and cerebellar symptoms or encephalitis have rarely been reported as a feature of MOGAD and represent a diagnostic challenge. Meanwhile, the coexistence of MOG IgG and other CNS autoimmune antibodies is infrequent. We report a patient presented with isolated epileptic onset, relapsed with MRI-negative brainstem symptoms and MRI-negative encephalitis. He was positive for MOG IgG throughout the disease course while concomitant NMDAR IgG was not detected positive until second relapse. He showed decreasing response to conventional first-line therapy. The last relapse was during a COVID-19 epidemic with limited inpatient resources. Fortunately, he was ultimately controlled on subcutaneous ofatumumab, a novel fully humanized anti-CD20 mAb. This is the first report about subcutaneous ofatumumab treatment in MOG and NMDAR IgG double positive encephalitis with 12-month follow-up, depicting its potential as a therapeutic option.

## Introduction

Myelin oligodendrocyte glycoprotein (MOG)- IgG–associated disorders (MOGAD) is a new entity comprising CNS demyelinating syndromes, mainly as acute disseminated encephalomyelitis (ADEM), optic neuritis, and myelitis. Isolated seizures and MRI-negative brainstem symptoms are rare presentations (1). Anti-NMDAR encephalitis (anti-NMDARe) is an autoimmune disorder characterized by prominent neuropsychiatric manifestations, seizures, dyskinesias, speech disturbances, and autonomic dysfunctions (2). Tough rare but NMDAR IgG and MOG IgG can exist in the same patient simultaneously or sequentially (3). The clinical and prognostic features may differ from those of single antibody disease. Ofatumumab is the first fully human anti-CD20 monoclonal antibody approved for relapsing multiple sclerosis that can be self-administered at home (4). The efficacy of ofatumumab against MOG IgG and NMDAR IgG double positive encephalitis is unclear. We report a patient of MOG and NMDAR IgG positive encephalitis who suffered relapses in short-term controlled on subcutaneous ofatumumab. He accepted four injections during 12-month follow-up without relapse or infection events.

## Case presentation

A 16-year-old male patient was admitted for slurred speech. He was seen in pediatrics 3 years ago for headache and a seizure attack after catching a cold. The seizure manifested as numbness and weakness of the left limb followed by twitching of the left limb with loss of consciousness for 5 min. Trauma, infection, poisoning, and family genetic history were denied. Repeated cranial MRI and MRA did not show significant abnormalities. CNS pathogenesis was negative ; EEG suggested right-sided slow wave changes. Cerebrospinal Fluid (CSF) analysis showed leukocytosis (62 leukocytes/uL and 95.3% monocytes), normal protein, and glucose level. Viral encephalitis was suspected, and he was treated with empirical antiviral therapy. No seizures occurred during hospitalization; he was discharged after a 1-week hospitalization.

Neurological examination suggested incomplete mixed aphasia, mild right-sided upper neuron facial palsy, right limb mildly paresis, and right-sided Bartholomew's sign positive. Routine blood count, biochemistry, and metabolic tests were between normal ranges. Serologic investigations (syphilis, hepatitis B, and HIV) and systemic autoimmunity were negative. CSF analysis indicated a normal opening pressure (165 mm H_2_O), lymphocytosis (90%, 6 leukocytes/uL), and normal protein and glucose level. The oligoclonal bands were pattern III, and pathological investigating including bacterial, fungal, acid-fast bacilli, cryptococci, and viral antibodies were negative. MRI plain and enhanced scans of brain and spinal cord were normal. ECG was unremarkable. Meanwhile, CSF and serum testings for MOG antibody and autoimmune encephalitis panel (antibodies against IgLON5, DPPX, GlyR1, DRD2, mGluR5, NMDAR, AMPA1, AMPA2, LGI1, CASPR2, GABAB, mGluR1, GAD65, Neurexin-3α, GABAA, KLHL11, AChR, AQP4, and GFAP) were conducted on a cell-based assay. MOG IgG were detected positive in serum (tilter 1:320) and CSF (tilter 1:100). He was diagnosed with MOGAD and was administrated intravenous methylprednisolone (1g/day, halving every 3 days). He achieved quick remission and was discharged.

Upon cessation oral prednisone himself, he was readmitted for neuropsychiatric symptoms 2 months later. Cognitive decline, choreiform movements, irritability, visual hallucinations, intermittent involuntary jerking, and weakness (4/5 MRC) of limbs were observed. Brain MRI was normal. CSF analysis indicated an elevated opening pressure (185 mm H_2_O), pleocytosis (14 leukocytes /uL; 85% lymphocytes), an elevated glucose concentration (5.4 mmol/L) (2.5–4.5 mmol/L) and a normal protein level. Anti-MOG antibodies were detected positive in serum (tilter 1:32) and negative in CSF. Meanwhile, anti-NMDAR antibodies were detected positive in serum (1:10) and CSF (1:10). He was diagnosed with MOG IgG and NMDAR IgG double positive encephalitis. IV methylprednisolone and IVIG (0.4/kg for 5 days) were initiated resulting mild remission so cyclophosphamide (1.5 g) was added, and levetiracetam was administrated to control limb shaking symptoms. His symptoms were gradually relieved, while memory impairment persisted. Levetiracetam and oral tapering prednisone were prescribed after discharge. However, he reappeared with irritability, auditory hallucinations and suspected seizures 40 days after discharge. The local regulations on quarantine against current COVID-19 epidemic caused limitation on admission, so the anti-CD20 new drug for multiple sclerosis ofatumumab (Kesimpta®) was suggested. He started to show remission on the third day of one injection of ofatumumab accompanied with oral tapering prednisone. No allergies or adverse reactions to injections occurred. He came for re-examination 20 days later. In addition to subtle tremors in both hands and memory deficit, his other symptoms disappeared. MOG IgG was detected positive in serum (tilter 1:32) and CSF (tilter 1:10), while NMDAR IgG was negative. The peripheral CD19 B- cell count decreased to 0 cells/uL (reference range, 90–560 cells/uL).

## Follow-up and outcome

**Figure 1:**
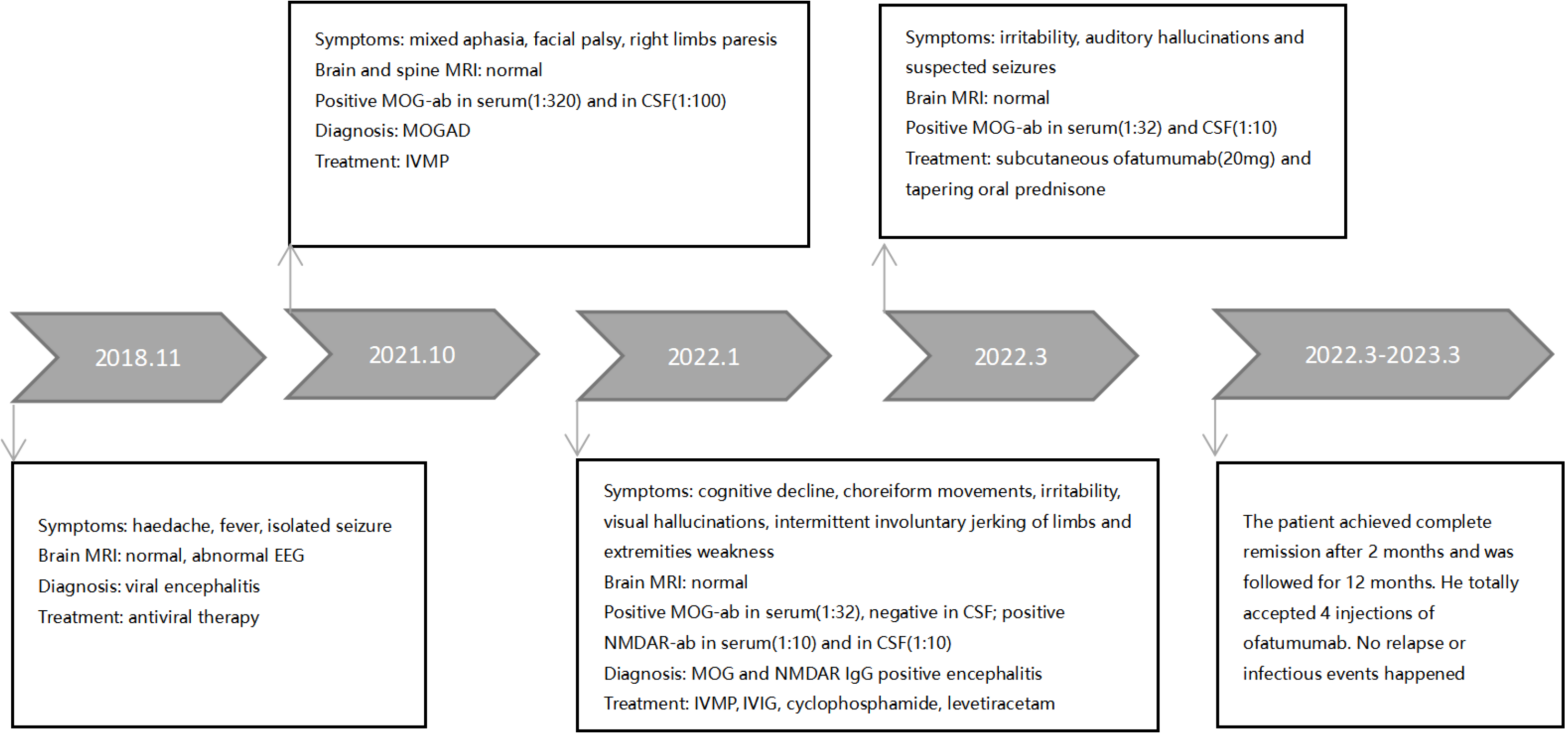
Timeline with diseases’ onset and course.

**Figure 2:**
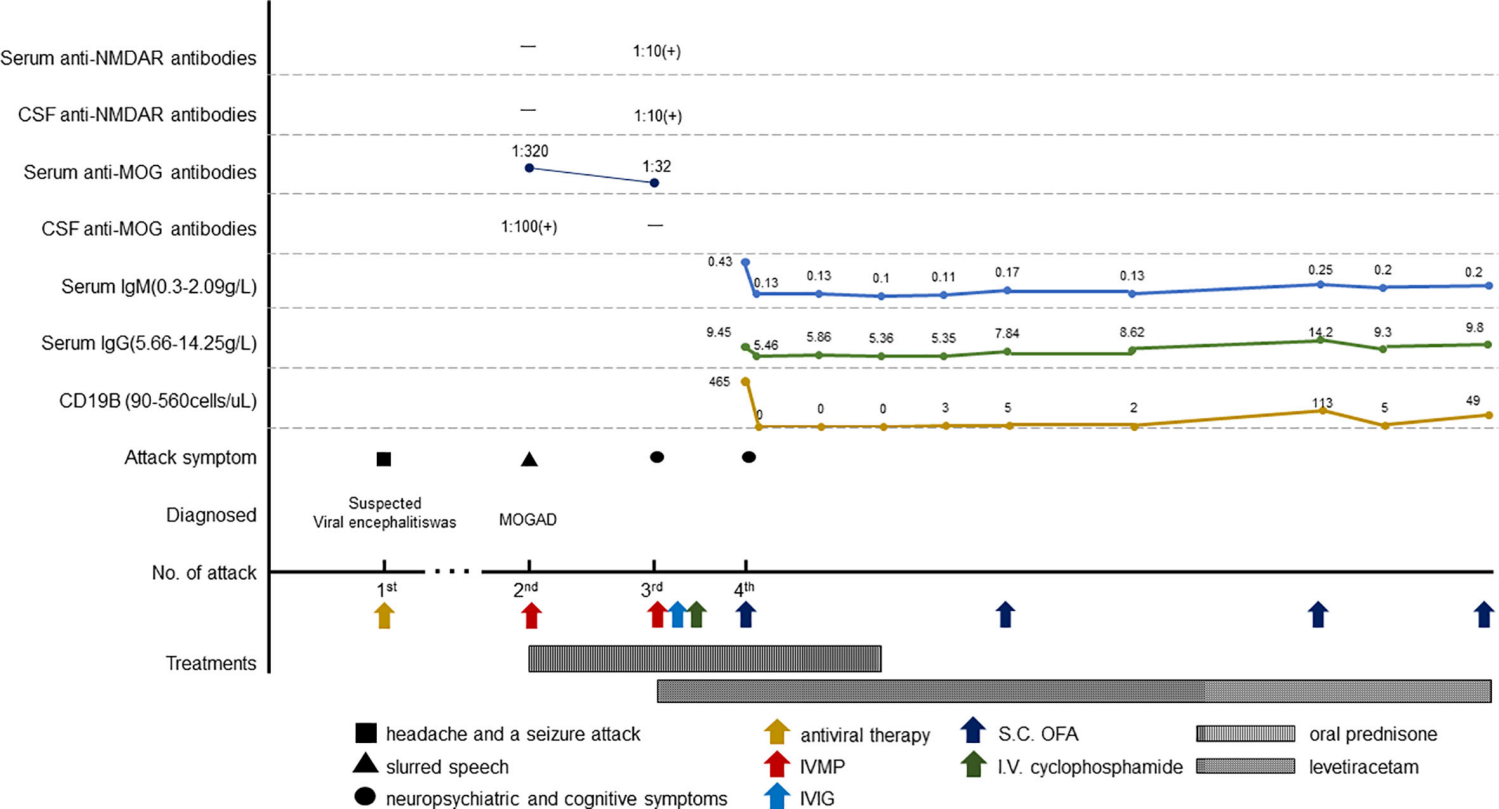
Schematic illustration of the disease course and follow-up. IVMP, intravenous methylprednisolone; S.C., subcutaneous; OFA, ofatumumab.

The patient achieved complete remission with no neurological defects remained at 2-month follow-up and achieved good academic performance (except for occasional pain in legs for prolonged glucocorticoid use). B-cell levels are measured monthly, and the infusion of ofatumumab is resumed when the CD19 B-cell count exceeds 10/uL. He accepted four injections of ofatumumab during 12-month follow-up. Blood IgM maintained stable levels. No relapse or infectious events happened until the recent follow-up and levetiracetam was discontinued. The clinical manifestations, important results of examinations, relevant diagnosis, interventions, and follow-up outcomes of the patient have been summarized in **Figures 1**, **2**.

## Discussion

As a relatively new spectrum of CNS autoimmune disorder, MOGAD not only shares features of multiple sclerosis and aquaporin-4-seropositive neuromyelitis optica spectrum disorder but also differs from them and is listed as a distinct disease entity (5). MOG- IgG–associated disorders comprise a wide spectrum of syndromes ranging from ADEM predominantly in children to optic neuritis or myelitis mostly in adults. The development and use of highly specific cell-based assays helps broaden the spectrum of MOGAD, with additional phenotypes including encephalitis, isolated seizures and overlapping syndromes with anti-NMDAR encephalitis, increasing its heterogeneity (1).

The incidence of seizures was found to be significantly higher in MOG-AD (20%) than in MS (6) (2.28%) and AQP4 antibody-positive NMOSD (7) (1%), so acute phase seizures are considered to be a unique clinical presentation of MOG-AD. Patients with cerebral cortical encephalitis and ADEM-like phenotypes are much more likely to develop symptomatic epilepsy than other phenotypes (8). Seizures can occur concurrently or sequentially with demyelinating events, or independently. Ramanathan (9) reported four MOG- IgG–positive children who first presented with isolated seizures without evidence of demyelination or cortical involvement, who months to years later developed typical demyelination, the exact mechanism of which is unclear. It has been speculated that MOG antibodies targeting oligodendrocytes with low density expression in the cortex are involved in the seizure mechanism, and it has also been suggested that a dramatic increase in inflammatory factors may be responsible for MOG-AD seizures (8). The low sensitivity of MRI may also affect the detection of early lesions. In this case, the child presented with isolated seizures 3 years before the onset of symptoms of encephalitis and unremarkable brain MRI. Due to the presence of prodromal infection and inflammatory CSF, CNS infection was considered preferentially despite negative pathogenic investigations. In retrospect, the presentation of isolated seizure may be part of the expanding clinical spectrum of MOGAD and was misdiagnosed. Testing for MOG antibodies acutely in children with seizures and inflammatory CSF manifestations helps with expedited diagnosis.

MOGAD tends to occur in children and mostly manifested as ADEM. MOG antibody encephalitis not fulfilling the criteria for ADEM was firstly reported by Ogawa (10) in 2017, although rare, relevant reports have garnered attention (11, 12). MOG antibody–associated encephalitis is well-circumscribed and cortical-based in neuroimaging, so it is called cortical encephalitis or MOG antibody-associated autoimmune encephalitis by relevant researchers (13). A large prospective multi-center observational study (14) showed that peadiatric patients with MOG antibody-associated encephalitis had clinical features including decreased level of consciousness (100%), seizures (64%), fever (59%), abnormal behavior (50%), motor deficits (40%), abnormal movements (36%), and brainstem–cerebellar dysfunction (23%). Brain MRI showed extensive cortical involvement, basal ganglia or thalamic involvement, in some cases also only minimal changes, or revealed normal scans. They also found that anti-MOG antibodies were more common than all neuronal antibodies among patients with autoimmune encephalitis with a proportion reached 34% (22 of 64). A study from China found that approximately 20% of patients had manifestations of encephalitis during the disease course (15). Predominant encephalic involves in younger patients more frequently, 93% of children had encephalitis symptoms at disease onset compared with 76.7% in adults (16). Cortical lesions in patients with MOG encephalitis and seizures are more apparent in FLAIR sequences. Such lesions are termed as FLAMES: FLAIR hyperintense lesions in anti-MOG encephalitis with seizures, which is thought to be the specific clinical phenotype of MOGAD (11).

Although MOG IgG is commonly associated with demyelination, negative MRI in MOGAD patients was not uncommon. In addition to the reported typical and atypical MRI features, the initial scan of the brain (14, 17, 18) as well as of the spinal cord (19) can be normal in patients with MOG- IgG–associated disorders despite severe clinical manifestation, which can lead to diagnostic uncertainty. It was reported that brain T2-hyperintense lesions are absent in 47%–68% of patients who have MOG-IgG (18). A Chinese cohort study identified two patients with MOG encephalitis in whom brain lesions were not visible on MRI but were confirmed by PET-CT (15). Our patient showed negative MRI throughout the disease course even with persisting MOG IgG positivity, which was very rare. Taken together, the clinical manifestations of MOGAD complex and variable, MOG IgG monitoring should be considered after ruling out infectious factors in these patients to avoid misdiagnosis.

MOG IgG may coexist with other auto-antibodies in patients with CNS autoimmune syndromes. NMDAR IgG is the most frequently associated antibody in MOGAD patients (3). It was reported that approximately 9% of MOG-IgG+ patients had CSF NMDAR-IgG+ (20). Meanwhile, 40.6% of MOG IgG–associated encephalitis patients had NMDAR IgG in CSF, and 16.4% had NMDAR IgG in serum (16). Patients with MOG and NMDAR IgG dual positivity can have presentations of both MOGAD and anti-NMDAR encephalitis, so this type disease is called MOGAD combined with anti-NMDAR encephalitis overlap syndrome (MNOS) (3). MOGAD and anti-NMDARe can happen simultaneously or sequentially in MNOS patients. A systematic review found that 44% (7 of 16) of patients were presented with anti-NMDARe at the onset of the disease, followed by 31% (5 of 16) were presented with MOGAD, and 25% (4 of 16) were presented with MOGAD and anti-NMDARe at the same time (21). Our patient had up to two additional relapses associated with persisting MOG-IgG when being detected positive NMDAR-IgG. There were similar cases reported before. Hou (22) reported one case developed anti-NMDAR encephalitis, 1 year after recovery from MOG- IgG–related ADEM. Wang (15) reported one patient exhibited a negative result during encephalitis but became anti-NMDAR-positive during a subsequent ON. Bojan (23) reported one case diagnosed with anti-NMDAR encephalitis nearly 12 years after four optic neuritis episodes without other signs of CNS demyelination. These reports demonstrate that MNOS is a syndrome with complex disease course. Encephalitis is the predominant clinical phenotype of MNOS, with convulsions, abnormal psychiatric behavior, sleep, and memory disturbances often being the first symptoms. Patients with MNOS usually present with supratentorial lesions and, in a few cases, with sub-tentorial and spinal lesions (20). The mechanism of MNOS is still unclear. The truth that a significant proportion of patients having signs of infection (headache and/or fever) prior to the onset of overlapping syndromes supports the molecular mimicry hypothesis (14). Considering that oligodendrocytes do contain NMDAR, it is reasonable to speculate that the immune attack targeting myelin may simultaneously involve NMDAR and vice versa. Meanwhile, some speculate that the use of immunomodulatory agents may trigger the generation of NMDA receptor or AQP4 or MOG antibodies or lead to immune dysregulation in overlapping syndromes (24). Most MNOS patients respond well to immunotherapy but have a tendency to relapse. Fan (21) reported five cases of children with MNOS, all of whom had relapses. Ren found that some MNOS patients were prone to relapse during hormone tapering, and that long-term drug maintenance therapy helped to reduce the disability rate (25).

As less common presentations are increasing with better recognition and availability of testing, recently the international MOGAD panel proposed new diagnostic criteria for MOGAD (1), which emphasize the presence of MOG-IgG as a core criterion. Patients with one of the core clinical attack types (including optic neuritis, myelitis, ADEM, cerebral monofocal or polyfocal deficits, brainstem or cerebellar deficits, and cerebral cortical encephalitis often with seizures) and clear positive MOG-IgG test results in serum measured by fixed or live cell-based assay can be diagnosed with MOGAD. Our patient presented with brainstem and cerebellar symptoms with strong positivity of MOG antibody after an isolated seizure attack, which meets the diagnostic criteria. The third episode was encephalitis with seizures with decreased response to treatment, which was more similar to the clinical of NMDARe rather than MOGAD, considering that most patients with anti-NMDAR encephalitis have normal, mild, or transient MRI abnormalities and nearly half of anti-NMDAR encephalitis patients need second-line immunotherapy (26).Moreover, the pathogenicity of MOG IgG in cortical encephalitis has been questioned. Ding (20) found that there was no difference in clinical symptoms between NMDAR-IgG+/MOG-IgG+ patients and NMDAR-IgG+/MOG-IgG− patients. Some scholars found no axonal and astrocyte damage and typical demyelination changes in the brain specimens of two patients with cortical encephalitis (27) and no increase in CSF- binding MBP in patients with cortical encephalitis in the case of extensive cortical involvement and CSF pleocytosis (10). Although disputed, it cannot be denied that encephalitis is a core clinical phenotype of MOGAD. The probability of double antibodies overlap may be underestimated due to changeable disease course. Anti-NMDAR antibody monitoring should be considered in patients presented with MOG-IgG associated encephalitis to avoid misdiagnosis, especially those with normal brain MRI scan, given that dual antibody does not always present simultaneously. The identification of dual antibodies helps guide to more aggressive acute treatment and maintenance therapy, resulting in a good clinical outcome.

Despite aggressive first- and second-line therapy, the patient experienced short-term relapses and showed reduced response to immunotherapy treatment. The last relapse was during a COVID-19 epidemic and the regulations against the epidemic resulted in limited access to hospitalization. On this condition, subcutaneous ofatumumab (20 mg) as first-line treatment was recommended and significant remission was achieved. This is the first report of ofatumumab treatment in a NMDAR-IgG/MOG-IgG double positive encephalitis. Ofatumumab is a fully human anti-CD20 mAb recognizing different epitope than rituximab (28). It is the first anti-CD20 drug that can be self-administered (subcutaneous injection) by patients and is approved for relapsing forms of Multiple sclerosis (MS) by the US Food and Drug Administration in 2020 (4). Ofatumumab has unique advantages of being the first fully human mAb that can be self-administered at home, which should result in less immunogenicity and more flexibility. This may be of particular relevance in the context of the current COVID-19 pandemic and any similar future situations. The recommended dosage for MS patients is 20 mg on days 1, 7 and 14 initially, then once monthly from day 28 (4). Since the last relapse happened in the midst of COVID-19 epidemic, taking account of the limitations of medical monitoring and the risk of increased infection from aggressive treatment, we have conservatively taken the approach of regularly monitoring B-cell levels to determine the timing of another injection. Fortunately, the refractory disease course with decreasing response to conventional immunotherapy (IV methylprednisolone, IVIG) showed favorable response to subcutaneous ofatumumab. The good attempt of ofatumumab in MOG and NMDAR IgG double positive encephalitis may indicate that ofatumumab is a convenient, effective, and safe option.

Limitations that need to be addressed as follows: (1) no accurate assessment of cognitive function in this form of encephalitis, (2) no repeated spinal MRI scans to look for underlying demyelinating lesions, and (3) lacks of several months’ follow-up data due to practical difficulties.

## Conclusion

MOGAD is a heterogeneous group of diseases with broad clinical manifestations, and isolated epilepsy may be its first manifestation. Testing for MOG antibodies should be included in patients with manifestations of encephalitis in which infectious factors have been excluded. In patients with MOG-associated encephalitis, especially those with recurrent episodes, attention should be paid to the presence of coexisting NMDAR antibodies. Noteworthy, subcutaneous ofatumumab may be a viable alternative option for CNS autoimmune diseases.

## Data availability statement

The original contributions presented in the study are included in the article/supplementary material. Further inquiries can be directed to the corresponding author.

## Ethics statement

Written informed consent was obtained from the individual AND minor’s legal guardian for the publication of any potentially identifiable images or data included in this article.

## Author contributions

RZ, XZ and BS treated the patient. LW and RZ drafted and revised the manuscript. KL and YT collected the data. YX designed the study and revised the manuscript. All authors contributed to the article and approved the submitted version.
